# The association between observed mobility and quality of life in the near elderly

**DOI:** 10.1371/journal.pone.0182920

**Published:** 2017-08-21

**Authors:** Jason Shafrin, Jeff Sullivan, Dana P. Goldman, Thomas M. Gill

**Affiliations:** 1 Precision Health Economics, Los Angeles, California, United States of America; 2 Schaeffer Center for Health Policy and Economics, University of Southern California, Los Angeles, California, United States of America; 3 Department of Internal Medicine, Yale School of Medicine, New Haven, Connecticut, United States of America; Nathan S Kline Institute, UNITED STATES

## Abstract

**Introduction:**

Chronic diseases associated with aging, such as arthritis, frequently cause reduced mobility, pain and diminished quality of life. To date, research on the association between mobility and quality of life has primarily focused in the elderly; hence, much less is known about this association in the near elderly. This cross-sectional study aimed to assess the association between mobility and quality of life measures in the near elderly.

**Methods:**

A prospective observational study of persons aged 50–69 years was conducted. The primary endpoint was quality of life measured by EQ-5D-5L, and the primary explanatory variable was observed mobility assessed using the 6-minute walk distance (6MWD). We applied regression models controlling for demographic, health status and other factors to evaluate the association between 6MWD and EQ-5D-5L.

**Results:**

Of the 183 participants analyzed in the study, 37% were male and the average age was 59.8 years. After adjusting for differences in demographic characteristics and health status, EQ-5D-5L-based utility values were 0.046 points (p<0.001), or 5.2% (95% CI: 2.7% to 7.8%), higher on average for individuals with 100 meters longer 6MWD. Holding constant the mobility-specific component of EQ-5D-5L, we still found that walking an additional 100 meters was associated with an EQ-5D-5L utility value that was 0.029 points (p<0.001), or 3.5% (95% CI: 1.7% to 5.5%), higher than the average participant. Among persons with arthritis, the association between 6MWD and EQ-5D-5L was slightly stronger.

**Conclusions:**

Near elderly persons with better mobility had higher quality of life. Diseases that decrease mobility, such as arthritis, are likely to have a significant impact on quality of life.

## Introduction

Mobility has been shown to be a key determinant of health and quality of life among the elderly.[1–4] Lack of physical activity has been shown to decrease cognitive function, reduce independence, and increase the risk of fractures, falls, and death.[5–11] Furthermore, elderly individuals who lose their mobility have been observed to have higher rates of morbidity, mortality, disability, hospitalizations, health care utilization and costs.[12–22]

The causes of limited mobility are multifaceted. On the one hand, a patient’s disease burden and number of comorbidities may affect the patient’s mobility. For instance, arthritis and osteoarthritis of the knee joint are common contributors.[11] On the other hand, a patient’s mobility is not simply the sum of separate disease processes; further, the relationship between anatomical or biochemical abnormalities, physical signs, and mobility function is often nonlinear in nature. [23]

Several studies have demonstrated that mobility is a key determinant of quality of life.[1–4] Using data from the LIFE pilot study, which examined a population of elderly adults at risk for disabilities, Groessl et al. found an association between mobility—measured by a 400 meter self-paced walk test—and quality of life—measured by the Quality of Well Being Self-Administered (QWB-SA) instrument.[1] Similarly, in a study of elderly Swedish adults, Fargerstrom et al. found that increased mobility improves individuals’ mental and physical quality of life.[2]

While prior research has largely focused on the association between mobility and quality of life in elderly persons (either age ≥ 70 years or age ≥ 60 years), [1–4] our study expands on previously published work by examining the association between observed mobility and quality of life in the near elderly (ages 50 to 69 years). Additionally, by measuring quality of life using the EuroQol-5 dimension (EQ-5D) instrument, we can translate the quality of life metrics into numerical utility weights, which enables broader comparisons to other diseases and health conditions.[24–26] Our study evaluates this association for all participants in the study as well as for the subset of participants with arthritis. Despite the well-known risks and consequences of reduced mobility, there has been limited research on the potential value of improving mobility in near elderly persons. To address this limitation, the current study evaluates the association between observed mobility and quality of life among persons aged 50 to 69 years.

## Methods

### Study population

This prospective observational study enrolled persons aged 50 to 69 years. Participants were required to be ambulatory and be able to walk for 6 minutes without sitting, using a walker, or the assistance of another person (use of a cane was allowed). Those with medical conditions that precluded safe participation in the 6-minute walk test were excluded in addition to those who were not able to provide informed consent. A sample size of at least 200 individuals was selected to achieve >80% power assuming an effect size of a 0.3% increase in quality of life from a 1 standard deviation increase in six-minute walk distance.

Potential participants were screened over the telephone to determine eligibility, confirm their age and provide demographic information, self-assessed ability to walk for six minutes, and history of joint replacements. The Callahan six-item cognition screen [27] was used to determine capacity to provide free and informed consent, and other questions were asked to determine the likelihood of successful participation in the study. Participants were then scheduled for a full assessment visit at the Yale Program on Aging. All participants who successfully completed the study were compensated for their time and effort. There was no follow-up contact of the participants.

Enrollment and data collection took place in the fall of 2015 and data were analyzed in 2016. Participants who underwent telephone screening to determine survey eligibility were required to provide oral consent to continue with the screening. Oral consent was documented through a verbal consent for telephone screening form where the telephone screener signed and dated each verbal consent provided. Participants who participated in the full survey were required to provide written informed consent to participate. IRB approval was obtained from Yale University’s Human Investigation Committee.

### Measures

The primary outcome was quality of life measured by the EQ-5D-5L survey instrument.[28, 29] The EQ-5D-5L is a self-reported questionnaire that describes respondents’ health using a descriptive system comprised of five items, each representing a different health dimension (mobility, self-care, usual activities, pain/discomfort, and anxiety/depression). For each dimension, respondents state whether they have no problems, slight problems, moderate problems, severe problems, or are unable to perform the activity. We then used estimates from a previous study that mapped responses of U.S. residents across all five EQ-5D-5L dimensions and translated these responses into a measure of a patient utility values or the value of a Quality Adjusted Life Year (QALY).[24–26] The utility values range from 1, which represents perfect health, to 0, which represents immediate death. [25] The EQ-5D-5L responses were scaled so that individuals who had no limitations across all five EQ-5D-5L dimensions were assumed to have perfect health. Quality of life was also examined using the EQ-5D visual analog scale (VAS) where respondents rate their quality of life on a continuous scale between 0 and 100, where 0 represents the “worst imaginable health state” and 100 the “best imaginable health state”.[25]

The primary explanatory variable of interest was 6-minute walk distance (6MWD). 6MWD is defined as the distance walked in 6 minutes without sitting and without the use of a walker or the help of another person (a cane may be used). The 6MWD is a well-established, valid and reliable measure of lower extremity performance in the elderly that has been used in a number of prior studies of mobility.[30–32] The walk was conducted in a wide hallway, specifically designed for this type of assessment, with a defined 20-meter course. The participants were instructed to walk as far as possible for 6 minutes at a speed they could maintain, and the distance covered was recorded.

Socioeconomic and demographic information was collected including characteristics such as age, gender, race, ethnicity, living situation, household composition, marital status, educational level, employment status, occupation, and income level.

Participants’ medical history, medications, and health care utilization were also assessed. The chronic conditions collected in the survey included arthritis, cancer, coronary heart disease or previous acute myocardial infarction, diabetes, hypertension, lung disease, and stroke. These diseases represent the seven physical health conditions collected in the Health and Retirement Survey.[33] Self-reported physical function was assessed in three areas: basic activities of daily living, instrumental activities of daily living, and general mobility.

### Statistical analysis

An ordinary least squares regression was conducted to evaluate quality of life as a function of 6MWD, participant demographics and socioeconomic status, and a series of health status indicators based on participant’s self-reported presence of each disease. Because arthritis directly affects joint pain and functionality, a sub-group analysis was performed on these participants. For both analyses, p-values were calculated using t-tests from this regression model.

Our analyses evaluated both the direct and indirect effect of observed mobility on quality of life. In our model, we measured overall quality of life using the EQ-5D-5L instrument, which includes a self-reported measure of mobility. The direct effect of improved mobility on quality of life occurs because participant mobility is one of the five dimensions included in the EQ-5D-5L measure. However, observed mobility could also have an indirect effect on four other dimensions of quality of life assessed by the EQ-5D-5L, which are depression, pain, and the ability of individuals to take care of themselves and do their usual activities. Thus, two analyses were preformed: in the first, the dependent variable was the observed EQ-5D-5L; and in the second, a rescaled EQ-5D-5L was used where the self-reported mobility component was held constant across all participants (the value for the mobility dimensions was set equal to the average value across all participants in our survey). The first regression captures both the direct and indirect effect of observed mobility, whereas the latter regression measures only the indirect effect of mobility on non-mobility quality of life factors. The predicted variable from both regressions can be interpreted as the expected quality of life for any 6MWD. As a sensitivity analysis to evaluate whether the results were sensitive to the survey instrument used, we repeated the baseline analysis using patient quality of life estimates reported using a visual analogue scale.

Participant characteristics included as independent variables in the regression model were demographic and socioeconomic factors and chronic conditions. The demographic and socioeconomic factors included age, gender, race/ethnicity, marital status, and education. The chronic conditions included arthritis, cancer, coronary heart disease or previous acute myocardial infarction, diabetes, hypertension, lung disease, and stroke. As a sensitivity analysis, we also examined the correlation between EQ-5D measures and 6MWD by age group (i.e., 50–54, 55–59, 60–64, and 65–69 years).

Statistical analyses were performed using Stata 14.1 software by Stata Corporation located in College Station, Texas.

## Results

A total of 287 individuals consented to participate in the study. Of these, 65 did not meet the inclusion criteria, 13 did not show up to the study after a phone consent, and 2 did not complete the six-minute walk test, leaving 207 individuals who completed the six-minute walk. Of these, 24 had missing or incomplete values for the EQ-5D-5L instrument. Among the 183 individuals included in the baseline analysis sample (Table 1), the average age was just under 60, and 37% of the population was male. More than 6 out of 10 participants (61.2%) were minorities, of whom 88.3% were African-American. The average 6MWD was 453 meters, and average EQ-5D-5L score was 0.886. Participants with arthritis were slightly older (61.1 years), less likely to be male, with somewhat less mobility (6MWD of 435.2 meters) and an average EQ-5D-5L score of 0.846.

**Table 1:** Characteristics of participants aged 50 to 69.

**Baseline Characteristics**	Mean (standard deviation) or n (%)	
	All Individuals (n = 183)	Individuals with Arthritis (n = 74)
**EQ-5D-5L**	0.886 (0.119)	0.846 (0.128)
**EQ-5D-5L (at average mobility levels)**	0.825 (0.079)	0.802 (0.094)
**EQ-5D Visual Analogue Scale**	86.9 (13.6)	82.1 (15.6)
**6-minute walk distance, in meters**	453.3 (81.0)	435.2 (80.0)
**Age**	59.8 (5.8)	61.1 (5.5)
**Male**	68 (37.2%)	24 (32.4%)
**Minority**	112 (61.2%)	46 (62.2%)
**Married**	67 (36.6%)	26 (35.1%)
**College or post college**	66 (36.1%)	28 (37.8%)
***Health status*:**		
**Arthritis**	74 (40.4%)	74 (100.0%)
**Cancer**	23 (12.6%)	10 (13.5%)
**Coronary heart disease^a^**	24 (11.5%)	10 (13.5%)
**Diabetes**	42 (23.0%)	24 (32.4%)
**Hypertension**	85 (46.4%)	43 (58.1%)
**Lung disease**	7 (3.8%)	4 (5.4%)
**Stroke**	5 (2.7%)	3 (4.1%)

Note

^a^ Coronary heart disease category also includes patients with a previous acute myocardial infarction

EQ-5D-5L, EuroQol-5 dimension-5 levels

### Association between mobility and quality of life

**Fig 1:**
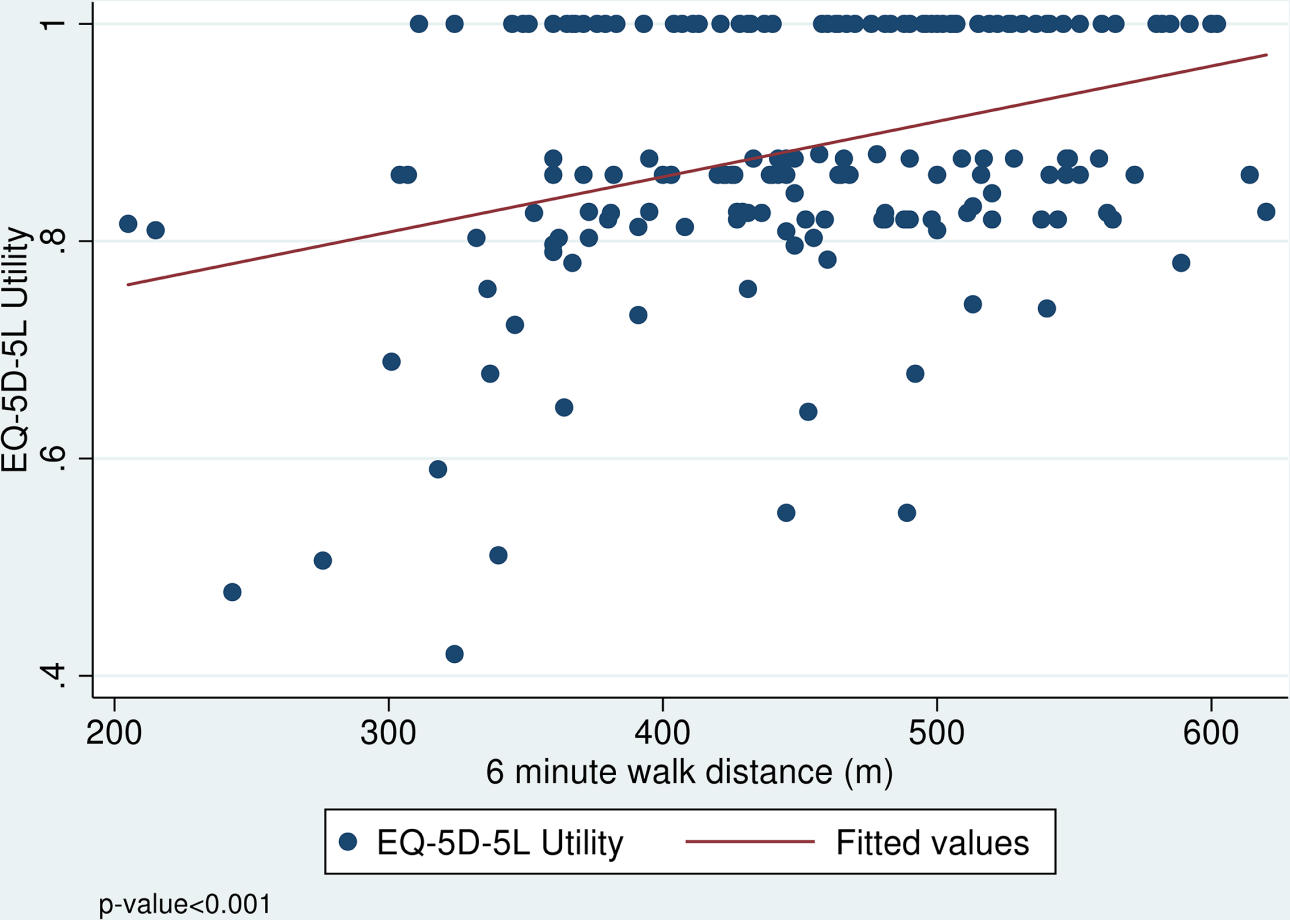
Scatterplot of 6-minute walk distance and EQ-5D-5L. EQ-5D-5L, EuroQol-5 dimension-5 levels; the lowest EQ-5D-5L utilities are calibrated so that a utility of 0 represents immediate death and EQ-5D-5L score of 1 is equivalent to full health.

For participants aged 50 to 69, a positive association was found between observed mobility and self-reported quality of life. In the scatterplot in Fig 1, a positive association is observed between mobility and EQ-5D-5L scores; participants who were able to walk longer distances in 6 minutes generally had higher quality of life. For each of the five dimensions of the EQ-5D-5L instrument, we observed that 6MWD was lower in participants with more functional impairment (S1 Table). We also observed a negative correlation (-0.455, p<0.001) between the 6MWD and the EQ-5D-5L self-reported mobility measure, indicating that participants who walked shorter distances in the six-minute walk test generally reported greater mobility impairments in the quality of life questionnaire. A similar relationship between mobility and quality of life existed among patients both with and without arthritis (S1 and S2 Figs).

**Fig 2:**
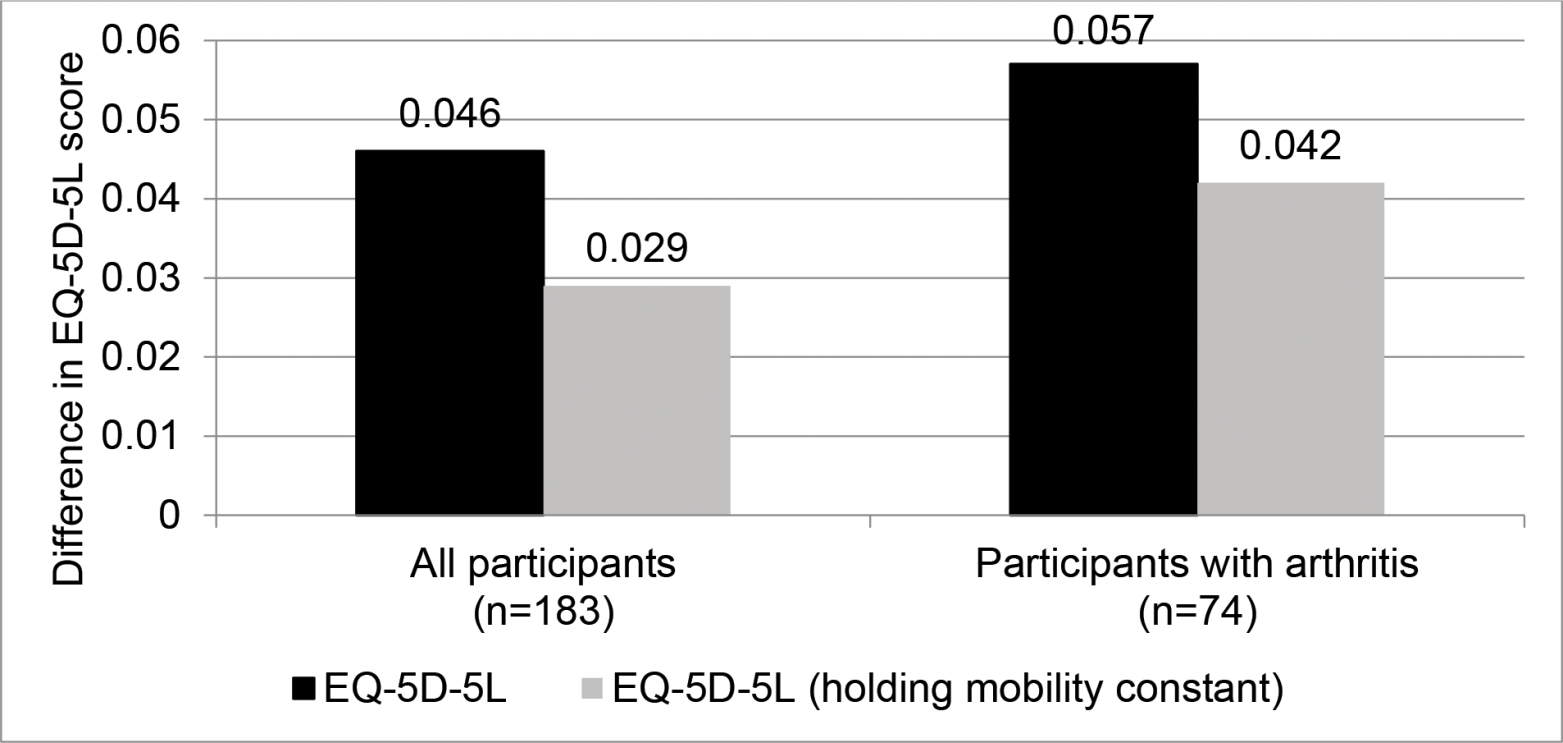
Difference in average EQ-5D levels for a 100-meter increase in 6-minute walk distance. EQ-5D-5L, EuroQol-5 dimension-5 levels; EQ-5D-5L score of 0 is equivalent to death; EQ-5D-5L score of 1 is equivalent to completely well. The difference in EQ-5D-5L score is measured based on the 100m difference in 6-minute walk distance as applied to our regression model.

After accounting for differences in participant demographics and health status, we found that better observed mobility was associated with higher quality of life. Fig 2 indicates that participants who walked an additional 100 meters had 0.046 higher EQ-5D-5L (p<0.001). This result corresponds to a 5.2% (95% CI: 2.7% to 7.8%) difference from the average participant’s EQ-5D-5L of 0.89. S2 Table displays the detailed regression analysis underlying the results presented in the figure. As shown in the table, 6MWD and quality of life have a statistically significant positive association. Because a majority of our sample was made up of racial minorities, we also tested whether the association between 6MWD and EQ-5D-5L utility varies by minority status. We found that higher mobility as measured using 6MWD is associated with a larger effect on EQ-5D-5L among minorities compared to non-minorities (p<0.05) (See S3 Table).

Similar overall results were obtained when EQ-5D-5L was estimated holding observed mobility constant. Patients who walked an additional 100 meters had 0.029 (p<0.001) higher EQ-5D-5L scores. This difference represents a 3.5% (95% CI: 1.7% to 5.5%) difference in average participants’ EQ-5D-5L score of 0.825.

The 6MWD had a larger significant positive association with the EQ-5D when it was measured using the visual analogue scale. Of the 207 individuals who completed the six-minute walk test, 15 did not report an EQ-5D-VAS value. Among these 192 individuals, walking an additional 100 meters was associated with an additional 6.2 points (p<0.001) in the EQ-5D-VAS. This difference indicates that these patients had a 7.2% (95% CI: 4.4% to 10.0%) increase in quality of life compared to the average participant’s EQ-5D-VAS estimate of 86.9.

Among the 74 participants with arthritis, higher levels of observed mobility were more strongly correlated with quality of life as compared to the full study sample. Participants who could walk an additional 100 meters in the 6 minute walk test had an EQ-5D-5L score that was 0.057 above the average participants’ score (p<0.01). Even when holding self-reported mobility constant, a positive association was found: an additional 6MWD by 100 meters in the 6 minute walk test corresponded to a EQ-5D-5L score 0.042 above that of the average participant (p<0.01). Similar results were obtained when EQ-5D was measured using the visual analogue scale, corresponding to a EQ-5D-VAS score of 8.5 points above that of the average participant (p<0.001). S4 Table summarizes results of the OLS regression for the arthritis population.

Additionally, we found a consistently positive association between quality of life and mobility across all age groups. The correlation between EQ-5D-5L utility and 6MWD was positive for each age group and statistically different from zero in two of four age groups. Using the EQ-5D-VAS, the correlation was positive across all four age groups, but was statistically different from zero in all four cases.

## Discussion

This study found that adults aged 50 to 69 with better observed mobility—as measured by 6MWD—had higher self-reported quality of life. While other studies have confirmed this association for adults ages 60 and above or 70 and above, this study focused on a population of near elderly adults. When also holding the self-reported mobility factor of the quality of life measure constant, a significant positive association existed between observed mobility and quality of life, indicating that improvements in mobility may have a positive impact on participants’ pain, anxiety, depression, and ability to complete their usual activities and care for themselves.

Based on current literature, the 5.2% difference in quality of life that was found to be associated with a 100 meter increase in 6MWD represents a non-trivial difference in quality of life. A 5.2% difference in quality of life is equivalent to the average quality of life difference—measured using EQ-5D-5L—among 40–49 year olds compared to 70–79 year olds, or the difference in quality of life among people with 2 chronic conditions compared to those with 3 chronic conditions.[34] In the context of specific chronic conditions, this difference is equivalent to the difference in quality of life between the average diabetes patient, and patients at low risk for diabetes. [35] Among those with chronic obstructive lung disease, this difference is comparable to the quality of life of patients with mild versus severe symptoms[36] and comparable to the difference in the median quality of life between those with advanced cancer that are considered fully active and those who are restricted in physically strenuous activity but ambulatory and able to carry out work of a light or sedentary nature.[37]

Previous studies also have observed a positive association between quality of life and mobility. A paper by Groessl et al., for instance, found that decreasing 400-meter walk time by 1 minute in older adults with mobility limitations increased overall quality of life by 1.3% above the average baseline estimate.[1] In a study measuring quality of life using the SF-12 metric, Fargerstrom et al. found that adults ages 60 and above who were not able to walk at least 400 meters without stopping were more than twice as likely to experience poor physical quality of life and more than 80% as likely to experience poor mental health compared with individuals who were able to walk 400 meters without interruption.[2] More generally, Oh et al. found that abnormal scores on the short physical performance battery (SPPB) instrument were associated with lower EQ-5D index levels. [38] Although we cannot prove that the association between mobility and quality of life is causal due to our cross-sectional study design, we did observe a strong, significant correlation. Other studies have also found that mobility, as opposed to other measures of physical function such as strength and balance, may be among the physical measures most closely associated with quality of life. For example, Sartor-Glittenberg et al. examined residents of a retirement community and found that gait speed, but not balance or strength, was associated with quality of life.[3] After adjusting for potential confounders, Trombetti et al. found a significant association between 400 meter walk time and quality of life measured by the physical component of the SF-36 scale in persons ages 70–85.[39]

### Limitations

This study had six primary limitations. First, the most severely ill persons—i.e., those who were institutionalized, wheelchair bound, or used a walker—were excluded from the study. Participant-reported quality of life in this study was higher than the national average of 0.84 for people ages 45–64.[40]

Second, these results should not be extrapolated to the national U.S. population. While 14.3% of the national population identity their race as African-American and 63% identify as Caucasian, 61.1% of the sample in this study identified as a racial minority. Our results found that the association between mobility and quality of life was stronger among racial and ethnic minorities compared to non-minorities. Additionally, our average 6MWD of 453 meters was less than the reported distances of 623 meters in a nationwide study of persons aged 60–64 and 591 meters of those aged 65–69.[41] These differences may be due to a higher prevalence of comorbidities in our study population relative to the aforementioned national studies. For example, 23% of participants in the current study had diabetes and 12.6% had cancer, values that are higher than those (18.9% and 9.3%, respectively) in the general population ages 55–64.[42]

Third, we only capture mobility using a single measure (i.e., 6MWD). The 6MWD is a well-established measure of lower extremity performance [30–32] that is commonly used to assess functional performance at a similar level required to perform daily activities.[43] Future research could aim to replicate this analysis using multiple mobility measures such as 400-meter walk time or steps per day to facilitate comparisons to other studies.[44, 45] Because the average 6MWD in our sample was 453 meters, however, we would not expect major differences between our results and a study that assesses 400-meter walk time. While a steps per day endpoint—measured either through a self-reported questionnaire or with a pedometer—has the potential to better capture the endurance rather than speed component of mobility, real-world steps per day endpoints suffer from the limitation that they measure both the individual’s physical ability as well as their lifestyle choices regarding how much mobility to engage in each day.

Fourth, self-reported quality of life measures are subject to numerous biases and may not fully capture participant quality of life.[46–48] The EQ-5D metric, however, is a well-studied measure often used by health technology agencies such as the National Institute for Care Excellence, and is also used as part of large national surveys such as the Medical Expenditure Panel Survey in the U.S.[49–51] Further, preferences among patients with mobility limiting diseases such as arthritis may differ from the general population in ways that are not reflected in the EQ-5D questionnaire. Prior research has found that arthritis patients value reductions in ambulatory pain, difficulty doing daily activities, pain while at rest, and stiffness as priorities,[52] however, the EQ-5D measure only assesses quality of life based on responses to a question about general pain rather than separating the contexts in which the pain occurs. Future research studying patients with osteoarthritis could collect a disease-specific measure such as the Western Ontario and McMaster Osteoarthritis Index to obtain quality of life measures among those whose mobility is more strongly associated with quality of life than the average near elderly person.

Fifth, this study measures only associations between patients’ 6MWD and quality of life. Since the study is not longitudinal, we cannot determine a causal or even a temporal relationship between mobility and quality of life. Future studies should aim to use longitudinal data to establish a temporal or potentially causal association between mobility and quality of life.

Finally, this study only measures the association between mobility and quality of life at the time of the assessment and does not provide insight into the impact of mobility on health economic outcomes over time. Future research may rely upon long run data or simulation models to estimate the impact of changes in mobility on various health economic outcomes such as health care spending, employment, and nursing home utilization.

## Conclusion

Near elderly persons with better mobility had higher quality of life. Diseases that decrease mobility, such as arthritis, are likely to have a significant impact on quality of life.

## Supporting information

S1 Data 6-minute walk distance data. (CSV)

S1 Fig Scatterplot of 6-minute walk distance and EQ-5D-5L among participants with arthritis. EQ-5D-5L, EuroQol-5 dimension-5 levels; the lowest EQ-5D-5L utilities are calibrated so that a utility of 0 represents immediate death and EQ-5D-5L score of 1 is equivalent to full health.
(TIF)

S2 Fig Scatterplot of 6-minute walk distance and EQ-5D-5L among participants without arthritis. EQ-5D-5L, EuroQol-5 dimension-5 levels; the lowest EQ-5D-5L utilities are calibrated so that a utility of 0 represents immediate death and EQ-5D-5L score of 1 is equivalent to full health.
(TIF)

S1 Table Mobility levels by EQ-5D response (all participants). Boldface indicates statistical significance. 6MWD, 6-minute walk distance. EQ-5D, EuroQol-5 dimension.
(DOCX)

S2 Table Association between mobility and quality of life^a^ (all participants). Boldface indicates statistical significance (*p<0.05, **p<0.01, ***p<0.001). ^a^ OLS regression with EQ-5D as the dependent variable. ^b^ EQ-5D-5L index values ranges from 0 (death) to 1 (perfect health). ^c^ EQ-5D Visual Analogue Scale ranges from 0 (death) to 100 (perfect health). ^d^ Coronary heart disease category also includes patients with a previous acute myocardial infarction. EQ-5D-5L, EuroQol-5 dimension-5 levels.
(DOCX)

S3 Table Association between mobility and quality of life^a^ (all participants, 6-minute walk distance and minority interaction). Boldface indicates statistical significance (*p<0.05, **p<0.01, ***p<0.001). ^a^ OLS regression with EQ-5D as the dependent variable. ^b^ EQ-5D-5L index values ranges from 0 (death) to 1 (perfect health). ^c^ EQ-5D Visual Analogue Scale ranges from 0 (death) to 100 (perfect health). ^d^ Coronary heart disease category also includes patients with a previous acute myocardial infarction. 6MWD, 6-minute walk distance. EQ-5D-5L, EuroQol-5 dimension-5 levels.
(DOCX)

S4 Table Association between mobility and quality of life^a^ (participants with arthritis). Boldface indicates statistical significance (*p<0.05, **p<0.01, ***p<0.001). ^a^ OLS regression with EQ-5D as the dependent variable. ^b^ EQ-5D-5L index values ranges from 0 (death) to 1 (perfect health). ^c^ EQ-5D Visual Analogue Scale ranges from 0 (death) to 100 (perfect health). ^d^ Coronary heart disease category also includes patients with a previous acute myocardial infarction. EQ-5D-5L, EuroQol-5 dimension-5 levels.
(DOCX)
