# Supplementary figures and images for: The association between observed mobility and quality of life in the near elderly
Source: PLoS One. 2017 Aug 21;12(8):e0182920. doi: 10.1371/journal.pone.0182920 (PMC5572211; doi:10.1371/journal.pone.0182920)

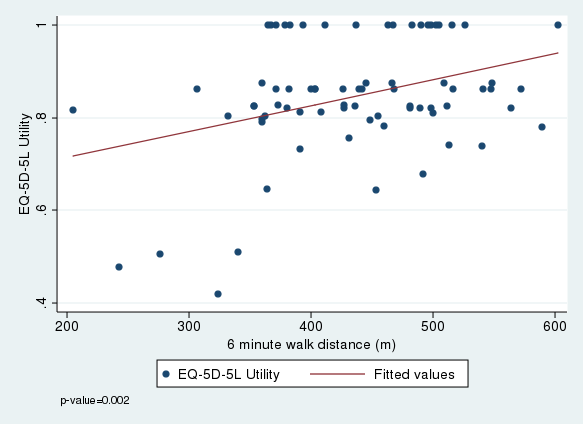

Supplement: S1 Fig — EQ-5D-5L, EuroQol-5 dimension-5 levels; the lowest EQ-5D-5L utilities are calibrated so that a utility of 0 represents immediate death and EQ-5D-5L score of 1 is equivalent to full health. (TIF) [file pone.0182920.s002.tif]

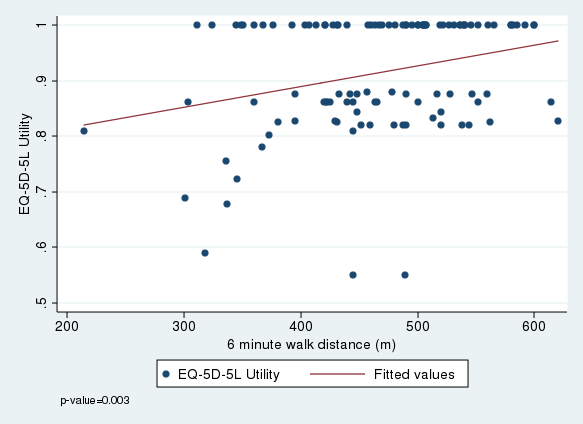

Supplement: S2 Fig — EQ-5D-5L, EuroQol-5 dimension-5 levels; the lowest EQ-5D-5L utilities are calibrated so that a utility of 0 represents immediate death and EQ-5D-5L score of 1 is equivalent to full health. (TIF) [file pone.0182920.s003.tif]
