# Supplementary material for: The association between observed mobility and quality of life in the near elderly
Source: PLoS One. 2017 Aug 21;12(8):e0182920. doi: 10.1371/journal.pone.0182920 (PMC5572211; doi:10.1371/journal.pone.0182920)
Supplement: S1 Table — Boldface indicates statistical significance. 6MWD, 6-minute walk distance. EQ-5D, EuroQol-5 dimension. (DOCX) [file pone.0182920.s004.docx]

S1 Table. Mobility Levels by EQ-5D Response (All Respondents)

|  | | **Usual Activity** | | **Anxiety/ Depression** | | | **Mobility** | | | **Pain/ Discomfort** | | | **Self-care** | | |  |
| --- | --- | --- | --- | --- | --- | --- | --- | --- | --- | --- | --- | --- | --- | --- | --- | --- |
| **EQ-5D Level** | **Description** | n | Avg 6MWD | | n | Avg 6MWD | | n | Avg 6MWD | | n | Avg 6MWD | | n | Avg 6MWD | |
| 1 | No problems | 158 | 464.9 | | 133 | 456.4 | | 146 | 470 | | 97 | 472 | | 172 | 459 | |
| 2 | Slight problems | 15 | 398.8 | | 39 | 448.4 | | 31 | 401 | | 64 | 447 | | 8 | 360 | |
| 3 | Moderate problems | 7 | 339.4 | | 8 | 444.6 | | 5 | 337 | | 19 | 404 | | 3 | 378 | |
| 4 | Severe problems | 3 | 385.0 | | 1 | 276.0 | | 1 | 243 | | 2 | 292 | | 0 | N/A | |
| 5 | Unable to walk | 0 | N/A | | 2 | 467.0 | | 0 | N/A | | 1 | 324 | | 0 | N/A | |
| Correlation (p-value) | | **-0.352** | **<0.001** | | -0.074 | 0.322 | | **-0.445** | **<0.001** | | **-0.336** | **<0.001** | | **-0.253** | **0.001** | |

Note: Boldface indicates statistical significance

6MWD = 6 minute walk distance; Avg = average; EQ-5D = Euroqol 5 dimension; N/A = Not applicable
