# Supplementary material for: The association between observed mobility and quality of life in the near elderly
Source: PLoS One. 2017 Aug 21;12(8):e0182920. doi: 10.1371/journal.pone.0182920 (PMC5572211; doi:10.1371/journal.pone.0182920)
Supplement: S2 Table — Boldface indicates statistical significance (*p<0.05, **p<0.01, ***p<0.001). a OLS regression with EQ-5D as the dependent variable. b EQ-5D-5L index values ranges from 0 (death) to 1 (perfect health). c EQ-5D Visual Analogue Scale ranges from 0 (death) to 100 (perfect health). d Coronary heart disease category also includes patients with a previous acute myocardial infarction. EQ-5D-5L, EuroQol-5 dimension-5 levels. (DOCX) [file pone.0182920.s005.docx]

| S2 Table. Association between Mobility and Quality of Life^a^ (All Respondents) | | | | | | |
| --- | --- | --- | --- | --- | --- | --- |
|  | **EQ-5D-5L Index^b^**  **(n=183)** | | **EQ-5D-5L Index^b^, holding self-reported mobility constant**  **(n=183)** | | **EQ-5D Visual Analogue  Scale^c^**  **(n=192)** | |
| **Covariates** | **Coefficient** | **95% CI** | **Coefficient** | **95% CI** | **Coefficient** | **95% CI** |
| 6-minute walk distance | **0.046***** | **(0.024, 0.069)** | **0.029***** | **(0.014, 0.045)** | **6.223***** | **(3.782, 8.663)** |
| Age | 0.002 | (-0.001, 0.006) | 0.002 | (-0.001, 0.004) | 0.144 | (-0.197, 0.486) |
| Male | -0.024 | (-0.058, 0.011) | -0.013 | (-0.037, 0.01) | -2.510 | (-6.319, 1.299) |
| Minority | 0.033 | (-0.004, 0.069) | 0.012 | (-0.013, 0.037) | 0.537 | (-3.447, 4.521) |
| Married | 0.033 | (-0.001, 0.067) | 0.023 | (0.000, 0.047) | 1.418 | (-2.362, 5.197) |
| College or post college | -0.027 | (-0.065, 0.011) | -0.020 | (-0.046, 0.006) | -0.616 | (-4.735, 3.502) |
| *Health status:* |  |  |  |  |  |  |
| Arthritis | **-0.046**** | **(-0.076, -0.015)** | **-0.023*** | **(-0.044, -0.002)** | **-5.310**** | **(-8.686, -1.934)** |
| Cancer | -0.050 | (-0.102, 0.001) | -0.022 | (-0.058, 0.013) | -3.187 | (-8.757, 2.384) |
| Coronary heart disease^d^ | -0.010 | (-0.056, 0.036) | -0.003 | (-0.035, 0.029) | -0.492 | (-5.659, 4.676) |
| Diabetes | -0.018 | (-0.058, 0.023) | -0.004 | (-0.032, 0.024) | -1.349 | (-5.836, 3.138) |
| Hypertension | -0.022 | (-0.058, 0.014) | -0.013 | (-0.038, 0.012) | 0.361 | (-3.608, 4.33) |
| Lung disease | -0.038 | (-0.108, 0.032) | -0.030 | (-0.078, 0.018) | -4.279 | (-12.135, 3.578) |
| Stroke | 0.020 | (-0.061, 0.100) | -0.010 | (-0.065, 0.045) | -1.986 | (-10.963, 6.991) |
| Constant | **0.561***** | **(0.348, 0.773)** | **0.619***** | **(0.474, 0.765)** | **53.139***** | **(29.85, 76.428)** |
| R-squared | 0.238 | | 0.188 | | 0.245 | |
| Note: Boldface indicates statistical significance (*p<0.05, **p<0.01, ***p<0.001)  ^a^ OLS regression with EQ-5D as the dependent variable  ^b^ EQ-5D-5L index values ranges from 0 (death) to 1 (perfect health)  ^c^ EQ-5D Visual Analogue Scale ranges from 0 (death) to 100 (perfect health)  ^d^ Coronary heart disease category also includes patients with a previous acute myocardial infarction  EQ-5D-5L, EuroQol-5 dimension-5 levels | | | | | | |
