# Supplementary material for: The association between observed mobility and quality of life in the near elderly
Source: PLoS One. 2017 Aug 21;12(8):e0182920. doi: 10.1371/journal.pone.0182920 (PMC5572211; doi:10.1371/journal.pone.0182920)
Supplement: S3 Table — Boldface indicates statistical significance (*p<0.05, **p<0.01, ***p<0.001). a OLS regression with EQ-5D as the dependent variable. b EQ-5D-5L index values ranges from 0 (death) to 1 (perfect health). c EQ-5D Visual Analogue Scale ranges from 0 (death) to 100 (perfect health). d Coronary heart disease category also includes patients with a previous acute myocardial infarction. 6MWD, 6-minute walk distance. EQ-5D-5L, EuroQol-5 dimension-5 levels. (DOCX) [file pone.0182920.s006.docx]

| S3 Table. Association between Mobility and Quality of Life^a^ (All Respondents, 6-Minute Walk Distance and Minority Interaction) | | | | | | |  |  |
| --- | --- | --- | --- | --- | --- | --- | --- | --- |
|  | **EQ-5D-5L Index^b^ (n=183)** | | **EQ-5D-5L Index^b^, holding self-reported mobility constant (n=183)** | | **EQ-5D Visual Analogue  Scale^c^ (n=192)** | |  |  |
| **Covariates** | **Coefficient** | **95% CI** | **Coefficient** | **95% CI** | **Coefficient** | **95% CI** |  |  |
| 6-minute walk distance | 0.021 | (-0.011, 0.053) | 0.010 | (-0.012, 0.032) | 2.941 | (-0.545, 6.426) |  |  |
| 6MWD*Minority | **0.046*** | **(0.004, 0.088)** | **0.034*** | **(0.005, 0.063)** | **5.892*** | **(1.36, 10.423)** |  |  |
| Age | 0.002 | (-0.001, 0.005) | 0.001 | (-0.001, 0.003) | 0.059 | (-0.284, 0.401) |  |  |
| Male | -0.027 | (-0.062, 0.007) | -0.016 | (-0.04, 0.007) | -2.915 | (-6.679, 0.849) |  |  |
| Minority | -0.176 | (-0.372, 0.019) | -0.144* | (-0.278, -0.011) | **-26.492*** | **(-47.648, -5.336)** |  |  |
| Married | **0.036*** | **(0.001, 0.07)** | **0.025*** | **(0.002, 0.049)** | 1.813 | (-1.922, 5.549) |  |  |
| College or post college | -0.024 | (-0.061, 0.014) | -0.018 | (-0.043, 0.008) | -0.092 | (-4.168, 3.985) |  |  |
| *Health Status:* |  |  |  |  |  |  |  |  |
| Arthritis | **-0.046**** | **(-0.076, -0.016)** | **-0.023*** | **(-0.044, -0.002)** | **-5.358**** | **(-8.683, -2.033)** |  |  |
| Cancer | -0.042 | (-0.094, 0.009) | -0.016 | (-0.051, 0.019) | -2.482 | (-7.995, 3.03) |  |  |
| Coronary heart disease^d^ | -0.012 | (-0.058, 0.033) | -0.005 | (-0.036, 0.026) | -0.752 | (-5.845, 4.342) |  |  |
| Diabetes | -0.014 | (-0.055, 0.026) | -0.001 | (-0.029, 0.026) | -1.052 | (-5.477, 3.373) |  |  |
| Hypertension | -0.019 | (-0.055, 0.017) | -0.011 | (-0.035, 0.014) | 0.745 | (-3.176, 4.666) |  |  |
| Lung disease | -0.050 | (-0.12, 0.02) | -0.039 | (-0.087, 0.009) | -5.771 | (-13.593, 2.052) |  |  |
| Stroke | 0.016 | (-0.064, 0.096) | -0.013 | (-0.068, 0.042) | -2.220 | (-11.063, 6.624) |  |  |
| Constant | **0.712***** | **(0.46, 0.964)** | **0.732***** | **(0.56, 0.905)** | **73.450***** | **(45.698, 101.202)** |  |  |
| R-squared | 0.258 | | 0.213 | | 0.271 | |  |  |
| Notes: Boldface indicates statistical significance (*p<0.05, **p<0.01, ***p<0.001)  ^a^ OLS regression with EQ-5D as the dependent variable  ^b^ EQ-5D-5L index values ranges from 0 (death) to 1 (perfect health)  ^c^ EQ-5D Visual Analogue Scale ranges from 0 (death) to 100 (perfect health)  ^d^ Coronary heart disease category also includes patients with a previous acute myocardial infarction  6MWD, 6-minute walk distance  EQ-5D-5L, EuroQol-5 dimension-5 levels | | | | | | |  |  |
