# Supplementary material for: The association between observed mobility and quality of life in the near elderly
Source: PLoS One. 2017 Aug 21;12(8):e0182920. doi: 10.1371/journal.pone.0182920 (PMC5572211; doi:10.1371/journal.pone.0182920)
Supplement: S4 Table — Boldface indicates statistical significance (*p<0.05, **p<0.01, ***p<0.001). a OLS regression with EQ-5D as the dependent variable. b EQ-5D-5L index values ranges from 0 (death) to 1 (perfect health). c EQ-5D Visual Analogue Scale ranges from 0 (death) to 100 (perfect health). d Coronary heart disease category also includes patients with a previous acute myocardial infarction. EQ-5D-5L, EuroQol-5 dimension-5 levels. (DOCX) [file pone.0182920.s007.docx]

| S4 Table. Association between Mobility and Quality of Life^a^ (Respondents with Arthritis) | | | | | | |
| --- | --- | --- | --- | --- | --- | --- |
|  | **EQ-5D-5L Index^b^**  **(n=74)** | | **EQ-5D-5L Index^b^, holding self-reported mobility constant**  **(n=74)** | | **EQ-5D Visual Analogue  Scale^c^**  **(n=76)** | |
| **Covariates** | **Coefficient** | **95% CI** | **Coefficient** | **95% CI** | **Coefficient** | **95% CI** |
| **6-minute walk distance** | **0.057**** | **(0.016, 0.097)** | **0.042**** | **(0.013, 0.071)** | **8.526**** | **(3.824, 13.228)** |
| **Age** | 0.002 | (-0.004, 0.009) | 0.002 | (-0.002, 0.007) | 0.247 | (-0.521, 1.015) |
| **Male** | -0.057 | (-0.124, 0.010) | -0.039 | (-0.087, 0.010) | -2.910 | (-10.841, 5.021) |
| **Minority** | 0.017 | (-0.053, 0.088) | -0.006 | (-0.058, 0.045) | -2.322 | (-10.536, 5.892) |
| **Married** | 0.042 | (-0.019, 0.102) | 0.039 | (-0.005, 0.083) | 2.856 | (-4.112, 9.825) |
| **College or post college** | -0.002 | (-0.070, 0.065) | -0.012 | (-0.061, 0.037) | 0.567 | (-7.340, 8.474) |
| ***Health Status:*** |  |  |  |  |  |  |
| **Cancer** | -0.072 | (-0.160, 0.017) | -0.050 | (-0.114, 0.015) | -9.186 | (-19.094, 0.722) |
| **Coronary heart disease^d^** | -0.014 | (-0.092, 0.063) | -0.010 | (-0.066, 0.047) | -0.572 | (-9.787, 8.644) |
| **Diabetes** | 0.005 | (-0.065, 0.075) | 0.016 | (-0.035, 0.067) | 2.700 | (-5.321, 10.721) |
| **Hypertension** | 0.020 | (-0.048, 0.088) | 0.010 | (-0.039, 0.060) | 2.729 | (-5.320, 10.778) |
| **Lung disease** | -0.038 | (-0.127, 0.052) | -0.041 | (-0.106, 0.024) | -6.322 | (-16.934, 4.291) |
| **Stroke** | -0.113 | (-0.272, 0.046) | -0.099 | (-0.214, 0.016) | -17.959 | (-36.617, 0.698) |
| **Constant** | **0.452*** | **(0.033, 0.872)** | **0.482**** | **(0.177, 0.787)** | 30.778 | (-17.356, 78.912) |
| **R-squared** | 0.271 | | 0.286 | | 0.297 | |
| Notes: Boldface indicates statistical significance (*p<0.05, **p<0.01, ***p<0.001)  ^a^ OLS regression with EQ-5D as the dependent variable  ^b^ EQ-5D-5L index values ranges from 0 (death) to 1 (perfect health)  ^c^ EQ-5D Visual Analogue Scale ranges from 0 (death) to 100 (perfect health)  ^d^ Coronary heart disease category also includes patients with a previous acute myocardial infarction  EQ-5D-5L, EuroQol-5 dimension-5 levels | | | | | | |
